# Predictive factors for failure of high flow nasal cannula among children with lower respiratory tract infection: A single center retrospective observational study

**DOI:** 10.1371/journal.pone.0328442

**Published:** 2025-07-14

**Authors:** Phanthila Sitthikarnkha, Rattapon Uppala, Apichaya Thiraratananukulchai, Prapassara Sirikarn, Leelawadee Techasatian, Suchaorn Saengnipanthkul, Sirapoom Niamsanit

**Affiliations:** 1 Department of Pediatrics, Faculty of Medicine, Khon Kaen University, Khon Kaen, Thailand; 2 Department of Epidemiology and Biostatistics, Faculty of Public Health, Khon Kaen University, Khon Kaen, Thailand; Jazan University, SAUDI ARABIA

## Abstract

**Background and objective:**

Lower respiratory tract infection (LRTI) is a leading cause of childhood hospitalization worldwide, with high-flow nasal cannula (HFNC) therapy commonly employed as a non-invasive respiratory support method. Determining early predictors of HFNC failure is crucial for timely intervention and reducing associated morbidity and mortality.

**Methods:**

We retrospectively reviewed the medical records of hospitalized children aged 1 month to 18 years with LRTI who received HFNC from January 2016 to December 2021. We aimed to evaluate predictive factors for HFNC failure and identify optimal cutoffs from the AUROC (AUROC).

**Results:**

One hundred and sixteen children with LRTI applied to HFNC were included in this study. Most of them (90.5%) were diagnosed with pneumonia as a principal diagnosis, and 68.9% had co-morbidities. Of those, 18 patients (15.5%) needed escalation to intubation and were defined as HFNC failure. The early predictive parameters associated with HFNC failure were pulse rate at 2 hours and SpO_2_/FiO_2_ ratio at 4 hours. AUROC analysis for the predictive parameter of HFNC failure showed cut-off values of S/F ratio at 4 hours after HFNC therapy was lower than 238 (AUC 0.89, 95%CI: 0.78–0.99).

**Conclusion:**

SpO2/FiO2 at 4 hours after initiating HFNC appeared to be a predicting value for early detected failure for HFNC therapy in children with LRTI. Close monitoring and swift management adjustments are recommended for children exhibiting these risk factors to enhance outcomes.

## Introduction

Lower respiratory tract infection (LRTI) is the leading cause of hospitalization in children worldwide [1]. While antiviral or antibiotic treatments are specific therapies for LRTI, oxygen support is crucial for managing acute dyspnea in hypoxic children [2]. Currently, various oxygen support methods are available, including nasal cannulas, face masks, non-invasive ventilation, and mechanical ventilation, which are selected based on the severity of hypoxia [3]. High-flow nasal cannula (HFNC) provides non-invasive respiratory support by delivering heated and humidified gas mixtures through a nasal cannula. HFNC affects the respiratory system and provides beneficial effects through several different mechanisms: (1) washout of the nasopharyngeal anatomical dead space, (2) reduction of inspiratory resistance by providing adequate flow, (3) improved mechanics by supplying adequate warm and humidified gas, (4) reduction in the metabolic cost of gas conditioning, and (5) provision of distending pressure [4].

HFNC therapy has recently been introduced as a safe and valuable therapy for hypoxic acute respiratory failure patients [5]. There is evidence that HFNC is an effective method for a range from infants to adults [6,7]. Its utilization is becoming more widespread, notably due to the enhanced comfort it provides pediatric patients compared to traditional respiratory therapy methods [8]. Additionally, HFNC is receiving heightened recognition for its effectiveness in managing pediatric patients in emergency department settings who present with acute respiratory distress [9]. It has demonstrated a significant reduction in the rate of intubation when compared to conventional oxygen therapy methods [10,11]. However, delayed intubation after HFNC failure leads to poor outcomes [12].

It is important to consider the factors that predict appropriate respiratory support for children. Early detection of HFNC failure may help physicians implement more advanced respiratory support measures earlier. Though the ratio of arterial oxygen partial pressure and fraction of inspired oxygen (PaO_2_/FiO_2_) has been suggested as a predictor for noninvasive ventilation in adults with acute hypoxemic respiratory failure [13], PaO_2_/FiO_2_ requires arterial blood gas sampling, which is difficult to collect in younger children or infants. The worsening of the Pediatric Early Warning Score (PEWS) at 90 minutes after HFNC initiation was observed in children who did not respond to HFNC therapy [14]. Nonetheless, this study focused on children experiencing respiratory distress from all causes. The respiratory rate-oxygenation (ROX) index has been reported to predict success in HFNC therapy among adults with pneumonia [15]. Recent studies have explored pediatric modifications of the ROX index (pROX) and ROX heart rate (ROX-HR) to account for varying respiratory rates with age and/or heart rate [16–18]. While these indices show promise, they are not yet universally adopted. Our study, therefore, centers on the SpO₂/FiO₂ ratio, which is feasible in a broad range of pediatric settings. The research focusing on children who experience respiratory distress from LRTI revealed that specific predictive factors for HFNC failure are scarce. Thus, this study aimed to identify the early predictive factors for HFNC failure in hospitalized children with LRTI.

## Materials and methods

### Study design and data sources

We performed a retrospective medical record review of hospitalized children aged 1 month to 18 years at Srinagarind Hospital from 1 January 2016–31 December 2021. This university-affiliated hospital is in the northeastern region of Thailand. Our study was conducted in adherence to the Declaration of Helsinki and was approved by the Khon Kaen University Ethics Committee for Human Research under the reference number HE641254. From 1 April 2022–31 December 2023, the data were accessed for research purposes.

### Study population

We conducted a study on children hospitalized for LRTI, including acute laryngotracheobronchitis, acute bronchiolitis, pneumonia, empyema thoracis, and lung abscesses. Our study focused on the children who received HFNC therapy due to respiratory distress from LRTI. We excluded children who had been intubated prior to admission, those using HFNC post-extubation, and patients with a tracheostomy.

### Respiratory management

At our medical center, the decision to use HFNC therapy for children with LRTI was made by pediatric pulmonologists. HFNC therapy was administered to children who exhibited tachypnea, increased work of breathing, or had pulse oximetry (SpO_2_) levels below 95% on room air. All children requiring HFNC therapy were admitted to the intermediate or pediatric intensive care unit (PICU). HFNC was delivered by high‐flow oxygen together with a blender and heat humidification system (MR850 heated humidifier, Fischer and Paykel Healthcare) or AIRVO2 (Fisher & Paykel Healthcare, Auckland, New Zealand). Fractional oxygen (FiO_2_) was adjusted from 0.21 to 1 to achieve SpO_2_ of at least 95%. Nasal cannulas were utilized with appropriate prong size, ensuring that the outer diameter occupied approximately 50% of the patient’s internal nasal diameter. The settings of HFNC therapy were adjusted by pediatric pulmonary and critical care staff. The flow rate and FiO2 for HFNC were adjusted according to clinical guidelines from the Royal Children’s Hospital Melbourne [19]. The initial flow rate started at 6 L/min and increased until continuous airflow was heard at basal lungs bilaterally. The maximum flow rate was calculated by body weight (kg). If the body weight was lower than 10 kg, the maximum flow rate was set at 2 L/kg/min. If the body weight was higher than 10 kg, the maximum flow rate was set at 2 L/kg/min for the first 10 kg and plus 0.5 L/kg/min for each kg thereafter (maximum flow 30 L/min). FiO_2_ started at 0.6 and titrated up to keep SpO_2_ greater than 95%. Apart from receiving respiratory support, the children were given standard treatment based on their individual conditions.

Transition to conventional oxygen therapy was recommended when the clinical condition had improved, as indicated by decreasing work of breathing and having normal respiration, and SpO_2_ greater than 95%. First, the FiO_2_ was weaned to 0.3 to keep SpO_2_ greater than 95%. Then, we further reduced flow by half; if the patients tolerated, then we changed to low-flow oxygen therapy.

HFNC failure was determined by the need for escalation to noninvasive positive pressure ventilation and endotracheal intubation with mechanical ventilation. This decision was based on the clinical criteria of respiratory failure defined including persistent or worsening hypoxia, increased work of breathing, and alteration of consciousness by pediatric pulmonary and critical care staff [20]. On the other hand, HFNC success was defined by improved respiratory symptoms after HFNC therapy and the patient’s ability to be liberated from HFNC.

### Data collection and outcome assessment

Data regarding age, sex, weight, height, diagnosis, underlying conditions, a pathogen of LRTI, complete blood count, and electrolytes were collected at the time of diagnosis. Respiratory rate (RR), pulse rate (PR), SpO_2_ were recorded by nurses at the time of starting HFNC and then at 1, 2, 3, 4, 8, 12, 16, 20, and 24 hours. Flow rate data and FiO_2_ were recorded simultaneously with vital signs. Because age‑specific expected respiratory‑ and pulse‑rate norms were not part of the bedside charts in use during 2016‑2021, composite paediatric indices that require these denominators (e.g., pROX, ROX‑HR, ROX‑M) could not be generated with methodological fidelity. The SpO2 per FiO2 (S/F) ratio is calculated by dividing the percentage of SpO2 by the FiO2 value, which ranges from 0.21 to 1.0, for the entire time period. The duration of HFNC usage was recorded as the time from initiation to successful liberation or escalation of HFNC. The length of the hospital stay was recorded from the admission date to the discharge date. In addition, we also collected data on the complications after using HFNC. All data from medical records were collected into Excel and subsequently transferred to Stata for analysis.

### Statistical analysis

All statistical analyses were conducted using Stata software version 15 (Stata Corp. 2017 Stata Statistical Software: Release 15, College Station, TX, USA: StataCorp LP.). Categorical variables are presented as numbers (n) with percentages (%). The normality of distribution was verified using the Shapiro-Wilk test. Continuous variables are expressed as median and interquartile range (IQR), or mean and standard deviation (SD), depending on the type of data distribution. We compared demographic data between children who failed and succeeded in using HFNC using chi-square or Fisher’s exact tests for categorical variables and independent T-test or Mann-Whitney U test for continuous variables.

To determine the predictive parameter, the generalized linear model evaluated PR, RR, SpO2, and S/F ratio at different time points. The complete case analysis was used to handle missing data. Predicted parameters associated with HFNC failure were evaluated with the area under the receiver operating characteristic curve (AUROC). The optimal cut point of the values was chosen to maximize the sum of sensitivity and specificity based on the receiving operating characteristic curves. A p-value of less than 0.05 was considered statistically significant. The 95% confidence interval (CI) was computed based on the normal approximation to the binomial distribution.

## Result

### Patient characteristics and treatments

**Fig 1:**
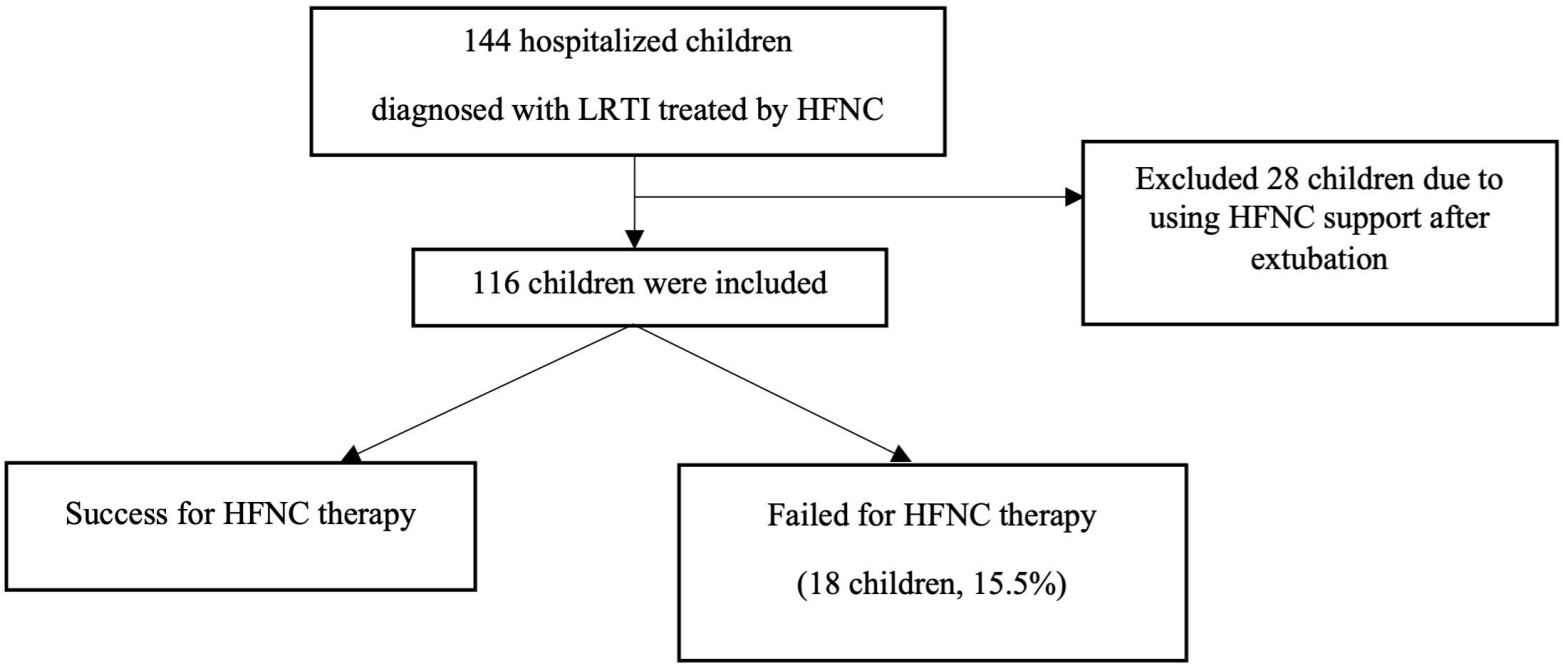
Study flow of hospitalized children with lower respiratory tract infection using high flow nasal cannula. LRTI; lower respiratory tract infection, HFNC; high flow nasal cannula, NIV; non-invasive ventilation, ETT; endotracheal tube intubation, IMV; invasive mechanical ventilation.

During the study period, 144 hospitalized children with LRTI were treated with HFNC. Twenty-eight children were excluded since they received HFNC support after extubation, leaving 116 children included in the study (Fig 1). Complete demographic data was available for all participants. Data on the complete blood count and serum electrolytes were missing in 3 (2.6%) and 2 (1.7%) children, respectively. Four children (3.4%) had partially missing SpO₂ or FiO₂ values at one or more time points, and 2 children (1.7%) had incomplete RR data. Beyond four hours after HFNC initiation, additional missingness reached 12% for RR and 18% for HR because recordings were charted at variable, non‑uniform intervals. These missing data were excluded from the respective analyses. The median age of the children was 26 (interquartile range [IQR] 12.5–54.5) months, and 56% were female. Pneumonia stood out as the predominant diagnosis, with acute bronchiolitis following closely behind at 90.5% and 6.9%, respectively. A total of 80 children (68.9%) had various comorbidities, with chronic respiratory disease affecting 22 children (19%) and neurological disorders impacting 19 children (16.4%), respectively.

In our study, 18 children (15.5%) were classified as HFNC failure, as they required an increase in respiratory support. Two patients were given non-invasive positive pressure ventilation, while 16 children needed to be endotracheally intubated with mechanical ventilation. Of this, the median time of escalation from HFNC in the failure group was 15 (IQR 7–24) hours. Children who failed HFNC therapy had higher age, body mass index, and comorbidities compared with the success group with statistical significance. The primary pathogen of LRTI in HFNC failure children was bacteria (16 children, 88.8%), while the success group was affected by viruses (48 children, 48.9%). Children with HFNC failure had a lower initial SpO₂ compared to the success group (95.4 ± 3.9% and 97.3 ± 3.1%, respectively; p-value 0.024). Nevertheless, there were no differences in FiO₂ between the two groups at HFNC initiation. Notably, upon initiation of HFNC, the children who experienced failure of HFNC therapy required a higher flow rate of HFNC (19.72 ± 7.96 LPM) compared to the success group (14.13 ± 5.98 LPM), with a p-value < 0.001. (Table 1).

**Table 1:** Characteristics of hospitalized children with lower respiratory tract infection using HFNC categorized by outcome of treatment.

Factors	Outcome of HFNC therapy		*p*-value
	Successful (n = 98)	Failed (n = 18)	
**Age (months), median (IQR)**	20.5 (10, 38)	28 (21.5, 38)	< 0.001
**Age group**			0.001
1 to < 12 months	27 (27.6)	1 (5.6)	
12 - 60 months	54 (55.1)	7 (38.9)	
> 60 months	17 (17.3)	10 (55.6)	
**Sex**			0.694
Female	55 (56.1)	11 (61.1)	
**Body mass index k(g/m** ^**2**^ **), mean ± SD**	15.5 ± 4.9	18.7 ± 5.2	0.015
**Indication of HFNC**			0.725
Pneumonia	88 (89.8)	17 (94.4)	
Bronchiolitis	7 (7.1)	1 (5.6)	
Croup	3 (3.1)	0 (0)	
**Pathogen**			0.002
Virus	48 (48.9)	1 (5.6)	
Bacteria	49 (50)	16 (88.8)	
Fungus	1 (1.1)	1 (5.6)	
**Comorbidity**	63 (64.4)	17 (94.4)	0.011
Chronic respiratory diseases	20 (20.4)	2 (11.1)	0.855
Neurological disorders	17 (17.4)	2 (11.1)	0.511
Cardiovascular disease	12 (12.2)	4 (22.2)	0.259
Hematologic disease	12 (12.2)	2 (11.1)	0.892
Genetic disease	8 (8.2)	4 (22.2)	0.072
Nephrological conditions	5 (5.1)	6 (33.3)	<0.001
Malignancy	4 (4.1)	3 (16.7)	0.039
Liver disease	4 (4.1)	0	0.383
**Initial flow (LPM), mean ± SD**	14.1 ± 6	19.7 ± 8	< 0.001
**Initial FiO**_**2**_ **, mean ± SD**	0.39 ± 0.07	0.39 ± 0.04	0.715
**Initial SpO**_**2**_ **(%), mean ± SD**	97.3 ± 3.1	95.4 ± 3.9	0.024
**Initial SpO**_**2**_ **/ FiO**_**2**_ **, mean ± SD**	265.0 ± 62.8	227.1 ± 67.8	0.012
**Initial RR (/min), mean ± SD**	43.7 ± 8.7	35.3 ± 6.3	< 0.001
**Initial PR (/min), mean ± SD**	145.6 ± 20.2	146.6 ± 21.0	0.842

Each parameter is expressed as number (%), otherwise presented as indicated.

HFNC; high flow nasal cannula, IQR; interquartile range, kg; kilograms, LPM; liter per minute, PR; pulse rate, RR; respiratory rate, SD; standard deviation.

### Predictors of HFNC outcomes

**Fig 2:**
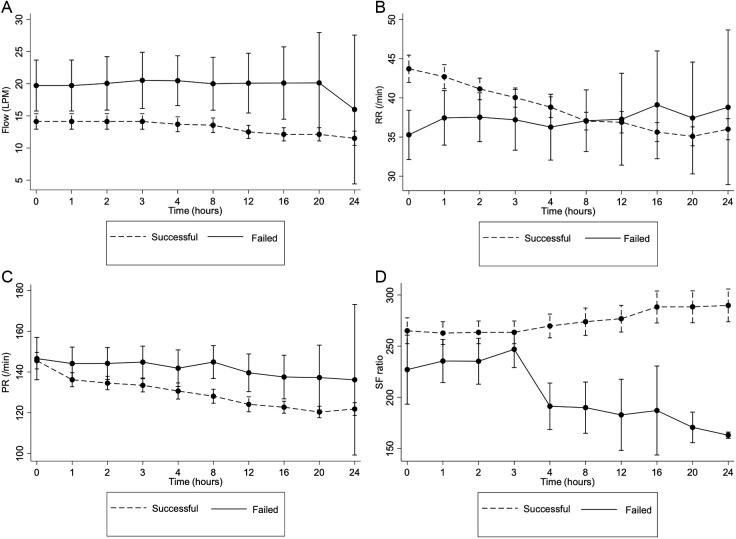
Changing of A. Flow, B. Respiratory rate (RR), C. pulse rate (PR), and D. S/F ratio of hospitalized children with lower respiratory tract infection after HFNC therapy categorized by the outcome of treatment. * Indicated p-value < 0.05. PR; pulse rate, RR; Respiratory rate, S/F ratio; SpO_2_/FiO_2_.

The flow rate of HFNC usage in the failure group was considerably high after 2 hours of HFNC initiation (Fig 2A). Although the RR in the failure group was lower at the start, it remained high after HFNC usage, whereas the success group had a lower RR after using HFNC (Fig 2B). There was no significant difference in PR at initiation in both groups, but the failure group had PR statistically higher than the success group after 2 hours of HFNC initiation (Fig 2C). The S/F ratio in HFNC failure was significantly lower at 4, 8, 12, 16, 20, and 24 hours of HFNC therapy (Fig 2D).

### Evaluating cutoffs of SpO2/FiO2 ratio for failure HFNC usage

Upon analyzing the ROC curve for the S/F ratio, it was determined that the S/F ratio appeared to have the highest diagnostic accuracy at 4 hours, with an AUROC of 0.89 (95% CI 0.78–0.99) with a cutoff level of less than 238. At this cutoff, the sensitivity and specificity were 95.9% and 73.3%, respectively (Table 2).

**Table 2:** Area under the curve of S/F ratio < 237.5 to distinguish failure from success of high-flow nasal cannula therapy at each time after initiation.

Parameters	AUC (95% CI)	Sensitivity	Specificity	Cut-off value
**S/F ratio at 2 hours**	0.76 (0.63 −0.90)	90.82	52.94	245
**S/F ratio at 3 hours**	0.75 (0.59 −0.90)	90.82	53.33	245
**S/F ratio at 4 hours**	0.89 (0.78 - 0.99)	95.92	73.33	238

### Outcomes of HFNC therapy

Among children in the HFNC failure group, two children (1.7%) died. They received HFNC therapy for 17 and 300 hours, respectively, prior to intubation, and both patients had comorbidities. The study found no complications from HFNC therapy. The length of hospital stays among children who failed HFNC therapy was higher than in the success group, accounting for 24 (13–41) and 5.5 (IQR 4–14) days (p-value < 0.001), respectively.

## Discussion

In this study of children hospitalized for LRTI who received HFNC therapy, we observed an HFNC failure rate of 15.5%. Among various physiologic parameters, the S/F ratio at 4 hours post-HFNC initiation emerged as a valuable predictor of failure, with a cutoff value lower than 238 associated with a higher likelihood of needing escalation to noninvasive or invasive ventilation. These findings underscore the importance of vigilant monitoring and timely intervention to prevent adverse outcomes related to delayed intubation. Our observed HFNC failure rate aligns with previous pediatric studies, which have reported failure rates ranging from 6% to 29.6% depending on patient selection, underlying diagnoses, and definitions of HFNC failure [21–24]. The median escalation time from HFNC in the failure group was 15 (IQR 7–24) hours, which was higher than the previous study [25]. Despite children in our study experiencing failure of HFNC therapy using higher flow, there were no documented cases of complications that could affect the treatment outcome.

The S/F ratio is based on easily obtainable clinical parameters, making it practical for use in clinical settings [18]. Recently, the S/F ratio was a reliable, noninvasive alternative for identifying and monitoring children with lung injury [26]. Previous studies have shown that lower S/F ratios were associated with a higher HFNC failure [21,18]. However, the previous study included children with heart failure [21] or children with heterogeneous respiratory problems [18], compared to our study which included children with respiratory distress from LRTI which had different pathophysiology from children with LRTI. An important finding in our study is that nearly 70% of children presented with one or more comorbidities, including chronic respiratory and neurological disorders. These conditions can significantly affect respiratory mechanics and the overall physiological reserve, thus requiring closer monitoring when initiating HFNC. Clinicians should remain vigilant about early signs of HFNC failure and be prepared to escalate support promptly in children with complex underlying conditions [22]. Patients in the HFNC failure group were older on average and required higher initial flow rates, which may reflect more severe disease or increased physiologic demands. These differences suggest that clinicians should closely monitor older children or those requiring higher flows, as they might have a higher propensity to fail HFNC. Interestingly, although the initial RR was lower in the failure group, it remained relatively elevated post-HFNC initiation, indicating persistent respiratory distress and a potential marker of impending failure. The cutoff of our study was similar as the previous research of children treated with HFNC due to acute hypoxic respiratory failure [27]. Even though the ROX index indicated the predictive factor for HFNC failure in adults, it is difficult to calculate in children with wide range of age group. The S/F ratio shows the feasibility of using it even in the limited resource hospital.

Our study emphasizes the S/F ratio as a 238 crucial predictor of HFNC failure, which has significant implications for the clinical management of pediatric patients with LRTI. By establishing a critical cutoff of less than 238 at four hours post-HFNC initiation, healthcare providers can identify at-risk patients early, facilitating timely interventions and potential escalation of respirator support to avert deterioration. Notably, prior research in heterogenous pediatric populations suggests that tools such as the ROX index, its pROX, or combined indices such as ROX-HR and the ratio of S/F to RR/median RR (ROX-M) might predict HFNC outcomes [16–18]. In our dataset, however, the requisite age‑adjusted RR and HR denominators and uniformly timed serial observations were absent or incomplete, precluding reliable reconstruction of these composite scores. We therefore selected the S/F ratio, which depends only on two values that were documented at every observation round and can be interpreted without age correction, as a pragmatic and easily standardised marker in resource‑limited settings.

Although the S/F ratio was a highly discriminative physiologic sign in our study, it should be seen as one component of a larger bedside assessment toolset available to nursing staff, who are closest to the patient and record respiratory observations hourly. Integrating the S/F ratio with validated early-warning systems, such as the PEWS, the Bedside PEWS, or pediatric adaptations of the ROX index (pROX, ROX-HR), may improve sensitivity for impending HFNC failure by coupling oxygenation data with dynamic trends in heart rate, respiratory rate, perfusion, and level of consciousness. Future protocols should include a multimodal nursing surveillance bundle that includes behavioral comfort, anxiety, and parental perceptions to enhance the generalizability and bedside utility of our findings across diverse pediatric settings.

Our study had some limitations. Firstly, this study was conducted in a single center which selection bias may limit the generalizability due to variation in HFNC protocol across different institutions. Secondly, it was a retrospective study; therefore, some data may be missing, such as the hourly record of work of breathing or retraction. However, the children in the study who used HFNC had to be admitted to the intermediate or intensive care unit, where their vital signs and HFNC settings were recorded hourly. Children were not randomized to HFNC vs. other modalities; hence, confounding by severity or underlying comorbidities may exist. We were unable to generate ROC curves or uniform thresholds for baseline RR, HR or flow because validated age‑specific reference values were not available in the retrospective charts. Thirdly, our study took place in a tertiary care center where most patients had underlying diseases that made them more prone to severe respiratory problems. Additionally, this study highlights the need for standardized protocols to identify patients with respiratory failure who require HFNC therapy and HFNC failure requiring intubation. Finally, most of the children in the study could not have arterial blood gas evaluated to meet the criteria for respiratory failure. However, the decision of respiratory failure was approved by pediatric pulmonary and critical care staff, who are experts in this area. Moreover, the absence of age‑stratified reference thresholds and the non‑uniform, partially missing nature of serial RR/HR recordings prevented calculation of paediatric ROX‑based indices, which we recognise as a significant limitation requiring prospective validation. Future prospective studies should incorporate such analyses to strengthen predictive modeling.

## Conclusion

In children with LRTI being treated with HFNC, the S/F ratio can be used to discriminate and predict treatment failure within 4 hours of starting HFNC, with a cutoff value lower than 238. Physicians should closely monitor the predictive factors for HFNC failure in the early stage of illness when deciding on HFNC therapy for children with LRTI.
